# Supplementary material for: A system analysis of the mental health services in Norway and its availability to women with female genital mutilation
Source: PLoS One. 2020 Nov 4;15(11):e0241194. doi: 10.1371/journal.pone.0241194 (PMC7641430; doi:10.1371/journal.pone.0241194)
Supplement: S1 Table — (DOCX) [file pone.0241194.s001.docx]

S1 Table 1. This is table 1. Informants and interviews

| **Individual informants** | **No. of persons**  **One interview** | **No. of persons**  **Two interviews** |
| --- | --- | --- |
| General Practitioners | 24 | 6 |
| Psychologists/psychiatrists | 9 | 3 |
| Gynecologists | 4 |  |
| Neurologists | 2 | 1 |
| Midwives | 19 | 4 |
| Psychiatric nurses | 8 |  |
| Sexologists | 3 |  |
| Administrative health personnel | 3 |  |
| **Focus group discussions** | **Health workers**  **(including Physicians, Nurses, Midwifes)** |  |
| Focus group 1: Health workers | 6 |  |
| Focus group 2: Medical Health Clinics | 7 |  |
| Focus group 3: Health Center | 4 |  |
