# Supplementary material for: A system analysis of the mental health services in Norway and its availability to women with female genital mutilation
Source: PLoS One. 2020 Nov 4;15(11):e0241194. doi: 10.1371/journal.pone.0241194 (PMC7641430; doi:10.1371/journal.pone.0241194)
Supplement: S1 Appendix — (DOCX) [file pone.0241194.s002.docx]

A semi-structured interview-guide for health-personnel at different levels and institutions.

Topics to be investigated, discussed and clarified

.

1. Information about the institution
   - Location, number of staff, what staff, number of patients, patient categories
   - Experiences with patients suffering from FGM/C
   - Experiences with patterns of mental health in different categories of population, immigrant women generally and women suffering from FGM/C or SSA women
   - Descriptions of the structure of referrals
2. Competence on FGM/C
   - What competence on FGM/C does the staff have?
   - Types of FGM/C, prevalence in different countries, within different ethnic groups
   - Consequences of FGM/C, physical and psychological consequences, short and long term
   - Competence and experiences with patients with FGM/C and treatments, duration and follow-ups
3. Patients

- Who are the patients that has problems with FGM/C
- Describe patterns of patients’ needs when suffering mental consequences of FGM/C
- Describe patterns of patients’ needs in general for SSA women
- Experiences of socio-sexual problems
- How do patients communicate their problems
- Challenges – diagnosis
- Expectations – disappointments – satisfactions
- Alternative treatment – home travel
- Somatization of mental health problems
- Referrals where

1. Thresholds, time, cost, availability

- Patterns of rejection and inclusions.
- How serious must the problem be for being referred and accepted
- Sign of mental health problems due to FGM/C
- The repertoire of health care for cut patients
- Communication and use of interpreters
- Alternatives for referrals –flow of referrals –challenges. Who goes where?
- The role of the municipality
- Who pays the treatment

1. Consultations

- What happens within a consultation
- Interaction
- Challenges
- Conduct understanding and challenges
- Feed back
- Other

1. Equal access to health care
