# Supplementary material for: A system analysis of the mental health services in Norway and its availability to women with female genital mutilation
Source: PLoS One. 2020 Nov 4;15(11):e0241194. doi: 10.1371/journal.pone.0241194 (PMC7641430; doi:10.1371/journal.pone.0241194)
Supplement: Attachment — Submitted filename: Response to reviewers.docx [file pone.0241194.s003.docx]

15/4 -2020

**Response to editor and reviewers**

We would like to thank the academic editor and the reviewers for their valuable time and feedback.

In this letter, we provide point-by-point response to both the editor and reviewers.

**Response to the academic editor**

1. Please ensure that your manuscript meets PLOS ONE's style requirements, including those for file naming. The PLOS ONE style templates can be found at <http://www.plosone.org/attachments/PLOSOne_formatting_sample_main_body.pdf> and <http://www.plosone.org/attachments/PLOSOne_formatting_sample_title_authors_affiliations.pdf>

Reply: Done

2. Please address the following:

a) Please ensure you have thoroughly discussed any potential limitations of this study within the Discussion section.

Reply: Done. Limitations and strength are added and discussed in the end of manuscript: line 681-693

b) Please include additional information regarding the survey or questionnaire used in the study and ensure that you have provided sufficient details that others could replicate the analyses. For instance, if you developed a questionnaire as part of this study and it is not under a copyright more restrictive than CC-BY, please include a copy, in both the original language and English, as Supporting Information.

Reply: Done. The methods section is rewritten. Line 202 -237

There is no questionnaire, but a semi structured interview-guide that contain topics to be discussed with informants. This interview guide is open and allows for issues to be raised during interviews.

The interview guide is added as supporting information S1 appendix

3. Your ethics statement must appear in the Methods section of your manuscript. If your ethics statement is written in any section besides the Methods, please move it to the Methods section and delete it from any other section. Please also ensure that your ethics statement is included in your manuscript, as the ethics section of your online submission will not be published alongside your manuscript.

Reply: Done

Following sentences are included in the Methods section: line 233 -238

4. Please ensure that you refer to Figure 1 in your text as, if accepted, production will need this reference to link the reader to the figure.

Reply: Done

5. Please include captions for your Supporting Information files at the end of your manuscript, and update any in-text citations to match accordingly. Please see our Supporting Information guidelines for more information: <http://journals.plos.org/plosone/s/supporting-information>.

Reply: Done. Added into supporting information is the following files.

S1 Appendix Semi-structured interview guide

S1 Table1 Informants and interviews

S1 Fig1 Socio-cybernetic model of mental health care

Reviewer # 1

1. The title is long. I suggest you delete this section: available to women………..

*Reply: We have shortened the title but must keep FGM/C in the title, thereby the title now sounds like:* *A system analysis of the mental health services in Norway and its availability to women with female genital mutilation*

2. Lines 66 -70 require references. References. Please see below article: Effect of female genital mutilation on mental health: a case- control study.

*Reply: Have included this suggested study together with 3 more references (6, 7, 8). Line 66-70, is now lines 82 -95*

3. Please write more detail about sampling (e.g. maximum variation or other type of sampling and why)

*Reply: The methods section is rewritten, and the purposeful sampling procedure is described.*

The method section is between line 202 -237

Semistructured interview guide added (S1 Appendix)

Table over informants and number of interviews added (S1 table 1)

Limitation and strength is added line 681-693

4. How many informants were interviewd twice?

*Reply: The method section is rewritten, and a table describing how many was interviewed once and twice has been attached. 14 informants were interviewed twice.*

*File added: S1 table 1 Informants and interviews*

5. Why “women suffering from mental health problems and FM/C” are not in interview list? And how do you analyze “the mental health services” without considering the customer’s viewpoint?

*Repy: This article focus particularly on the health workers perspective on their own system and on the patient groups they serve, which is a valid perspective. The overall project has several modules, and interviews with Gambian, Eritrean and Somalian women have been done to examine their perspectives. Results from these interviews will be published in a separate article that will cover perspectives of both health workers and these user group on equal access to health care.*

*A new background information paragraph (line54 -62) is introduced in the beginning to inform that this article only represent a part of the whole project.*

6. This sentence has been repeated twice. “ Some informants were interviewed several times and were used as key informants. (lines 162 and 179)

*Reply: Removed repeated sentence 179. To better describe the informants who were interviewed twice, a table is introduced showing the different health workers and who were interviewed twice. Added file in supporting information (S1 table1)*

7. Please add the methods of analysis and coding of interviews.

*Reply: an anthropological field work method require that data is analyzed during the data collection process itself based on the analytical tools that is provided by the theory which in this case is socio cybernetic theory. Described in line 231 and 232. This theory has been presented and discussed in the Findings and discussion section in relation to data.*

8. This is part of the findings (not a separate section) “ A system of mental health and its borders and entries.

Reply: The section is included in the Findings and discussion section.

Reviewer # 2.

Reviewer # 2: Abstract: I think sub-headings are required in the abstract. The abstract implies that the focus on the paper is with data from a sub-sample of GPs. However this is not the case. Hence, I think the abstract needs to be re-written a little to reflect the actual paper.

*Reply: Done. Abstract is rewritten* with sub-headings. Line 23 - 50

GENERAL COMMENTS

Reviewer # 2:Two key assumptions need to be examined more and explained. The first is that because women are not referred for psychological help, specifically for FGC related issues, the system has failed them.

*Reply: Nowhere is it written that the system fails the SSA women, but that the system diverts and redirect patients towards services at the municipal level. This is a big difference, and this is captured by the socio-cybernetic analysis. As the reviewer said: “centers at this level may receive patients in a cultural appropriate way”. .*

*It may be that the term “bottom” is misguiding and indicating of failure, thereby having a moralistic undertone. “The bottom” is in many places replaced by “services at the Municipal level”, in order to avoid a moralistic undertone and misunderstanding.*

*However the article show that the “specialist services” is not easily accessible for many of these women with mental health problems, which is a weakness of the system,* thereby the patients are directed to other parts, the municipality level which is shown by the cybernetic analyses. The concept ”bottom” is also removed from the figure (S1 fig 1).

Reviewer # 2: There is not any strong evidence presented that women with FGC actually want or need psychological help specifically for FGC-related issues. By saying this, I am not denying that they may need help for FGC-related issues or for other traumatic issues or other psychological needs, but I am suggesting that the authors need to create a much stronger argument to justify their statements that women from Somalia/Eritrea need psychological help specifically for FGC?

Reviewer # 2: “However, there is not any strong evidence presented that women with FGC actually want or need psychological help specifically for FGC-related issues”.

*Reply: More references on this issue has been suggested by reviewer 1 as well. Three more studies and a study review are introduced in the line 79-95, presenting evidence that there is a link between FGM/procedure, PTSD and depression, thereby indicating a need for mental health services. Research has also shown that women themselves not necessarily link their psychosexual problems or physical problems to FGM/C. This indicate that mental health problems and needs must be discovered in consultations, the same way that heart failure or diabetes can be discovered when a patients goes for consultation related to another health problem, or does not understand that there is a health problem at all when a doctor discovers it. Unexpected discovering by the GP requires time available. Lack of time has been discussed in the article.*

Reviewer # 2: Where is the evidence from women with FGC showing that they need FGC-specific psychological support? This evidence needs to be cited.

Reply: *Evidence is cited and discussed from line79 to 95*

*In many studies it is shown that women themselves see FGM as contributing to health benefit rather than the opposite, and that these conceptions together with other conceptions contribute to the maintenance of the tradition rather than abandoning it . (Berg et al. 2010). This information is cited in line 79 to 95.*

Reviewer 2: In addition, it is interesting that the data show that the professionals in the study who currently work with these women do not seem to state that they need it. Nor do they give personal examples of any experiences with women who want psychological help specific to FGC.

Reply*: Correct. This is the finding from the research. The women as well as GPs and other health workers are silent about the issue, Neither the GPs nor the women link mental health problems to FGM/C even though there may be a link.*

Reviewer # 2: Hence, it currently feels like the argument in the paper is proceeding based on an *assumption that FGC-specific psychological help is an important issue.* I think this assumption needs to be more thoroughly evidenced, problematised and discussed – both in the background section and then again in the discussion.

*Reply: Done. Evidence is provided in the litterature. The link between FGM and mental health is strengthened and discussed in the background section. Line 79 -95. It indicates that psychological help is an important issue.*

Reviewer # 2: The second assumption is the implied criticism of the system because women from SSA access help through the health life centers. In fact, the data seems to suggest that these centers are meeting needs in a very culturally appropriate way – by providing a way into care through a focus on physical health, but then by providing access to other services and, importantly, a community where women feel safe and able to talk. Yet, the authors are referring to them as being at the ‘bottom’ of the system. On what basis is this criticism made? Perhaps in fact, more centers such as this need to be set up and supported?

*Reply: The system is hierarchical and organized in levels, and the municipality level lies at the bottom, which is shown in the model. This conceptualization “the bottom”is not a criticism of the model. The article shows where the patient flow goes. The municipality institutions are fairly new and they are supposed to represent low threshold at the lower level of the system .However the term “bottom “ is substituted with the term “ municipal level” in most places in order not to sound or stress a moralistic perspective. However, there is a problem for patients to get access to the specialist services, which is a finding from the study, and they mostly are directed to the Healthy Life centers rather than the other services at the municipal level, which could also have benefitted them as the reviewer indicates. .*

Background

Reviewer # 2: Lines 84-88 (explaining levels of mental health amongst Somalis and Eritreans) – uses colloquial language (“described as a mystery”) – please re-word

*Reply:* The authors does not call it a mystery, they refer to a study where a particular Norwegian researcher describes his finding as a mystery. He also indicates that Somalis underreport their problems. In line 108 -109, the word “mystery” is linked to the researcher who did the study.

The concept mystery is kept, as it is cited from a research report.

Reviewer # 2: A key issue in this section is that it is slightly unclear why the authors have chosen to focus upon mental health when they have described that levels of mental health among Somali/Eritrean women appear to be similar to the general population (i.e. the case for the focus on mental health needs to be stronger)?

The researcher who presented these results, suggest an interpretation of the result as underreporting, which is referred in this study from Norway. Studies from the Netherland, and other places, shows different results. These studies showing that FGM has a negative effect on mental health, have been cited earlier, line 82 -95

.

Reviewer 2: In several places, the term “i.e.” seems to appear inappropriately (lines 93, 94)

*Reply: i.e. is removed from lines 93, 94*

Reviewer 2: The sentences in the paragraph in lines 93-101 seems rather poorly constructed. I would suggest re-writing. Does it need a sub-heading “Research Aim”?

*Reply: The paragraph has been rewritten. Now line 125-136*

Reviewer # 2: The background provides a brief description of the organisation of the GP system in Norway. I would suggest that the whole ‘mental health’ system needs to be more clearly and properly explained.

*Reply: The system is described under the heading Findings and discussion, where the model is presented in (Fig 1). New lines addressing the referral rules is added from line 255 to line 261. Further the low threshold services are described from 260 -271*

Reviewer # 2: The section on the health system as a ‘socio-cybernetic system’ needs a bit more elaboration, including more examples of how and when and why it has been used in healthcare. I think there also needs to be more explanation as to why this approach was chosen in contrast to other possible approaches? What new insights can be gained by using this approach?

*Reply: There are examples already presented of where the cybernetic system analysis of the health system has been done. However the benefit of this approach is presented, in line 185 – 194.*

*The benefit of a cybernetic analysis is also described in the Conclusion 632-636*

A SYSTEM OF MENTAL HEALTH AND ITS BORDERS AND ENTRIES

Reviewer # 2: The section in lines 231-242 about Somali’s health seeking behaviour would be better placed in the Background section as part of the rationale for doing the study.

Reply: This part has been taken out and put in the background section. Added: line 113- 124

Reviewer # 2: The finding about the GPs referral letters not being accepted is interesting but needs more explanation. As a non-Norwegian, I can’t understand why another service would reject a referral. Did the GPs give any examples of women from SSA who they had referred for depression but whose referral had been rejected?

Reply: More explanations on the referral system is presented in line 255 -261

Reviewer # 2: The section on ‘health centers’ (lines 325-339) suggest that midwives see more patients with FGC than GPs (or discuss it more openly). The authors note that midwives do not have referral right to send a patient for psychological care. However, the paper does not make it clear whether or not the midwives would like to have this right or whether they feel they need it. What did the midwives have to say about women with FGC and depression?

*Reply. The Health Centers is related to the health needs of children, youth and pregnant women, and does not have referral rights to gynecologists and psychologists, but they register pregnant women so that a place will be arranged for them when they are going to give birth at the hospitals.*

*The midwives do not have referral rights and we do not have data on whether they would like to have it or not. As is shown in the article, the topic of mental health and FGM is not on the agenda at the Health Centers.*

Reviewer # 2: This section should be re-titled ‘Discussion’ and should relate the findings to the wider literature and policy. The section needs to include recommendations for policy and future research? In addition, it should include a discussion on the value of having utilised a socio-cybernetic system approach*.*

*Reply: We have maintained the conclusion but have moved discussions to the heading: Findings and Discussion.*

*In conclusion we have summarized the findings in line with the socio-cybernetic analytical concepts, and we have come up with policy recommendation, and recommendation for further research: line 664 – 780*

*The value of a cybernetic analysis is presented in the conclusion line 632 – 634 and line 677 – 680*

Yours sincerely,

Inger-Lise Lien

PhD in Social Anthropology, Research Professor, Researcher 1 at NKVTS

Section for Trauma, catassstrophes and forced Migration

Norwegian Centre for Violence and Traumatic Stress Studies (NKVTS)

[i.l.lien@nkvts.no](mailto:i.l.lien@nkvts.no) (ILL)

Yours sincerely
